# Shedding new light on the context and temporality of Iberian warrior stelae: The Cañaveral de León 2 Stela and Las Capellanías burial complex (Huelva, SW Spain)

**DOI:** 10.1371/journal.pone.0321080

**Published:** 2025-04-23

**Authors:** Leonardo García Sanjuán, Timoteo Rivera-Jiménez, Marta Díaz-Guardamino, David Wheatley, José Antonio Lozano Rodríguez, Teodosio Donaire Romero, Antonio César González-García, Raquel Montero Artús, José Ruiz Flores, Javier Bermejo Meléndez, Miguel Ángel Rogerio-Candelera, Johan Ling, Eric Andrieux, Ian Bailiff

**Affiliations:** 1 Department of Prehistory and Archaeology, University of Seville, Seville, Spain; 2 Department of Archaeology, Durham University, Durham, United Kingdom; 3 Department of Archaeology, University of Southampton, Southampton, United Kingdom; 4 Spanish Institute of Oceanography, CSIC, Santa Cruz de Tenerife, Spain; 5 Department of Earth Sciences, University of Huelva, Huelva, Spain; 6 Institute of Heritage Sciences (Incipit), CSIC, Santiago de Compostela, Spain; 7 Department of History, Geography, and Anthropology, University of Huelva, Huelva, Spain; 8 Institute of Natural Resources and Agrobiology of Seville, CSIC, Seville, Spain; 9 Department of Historical Studies, Swedish Rock Art Research Archives, University of Gothenburg, Gothenburg, Sweden; Austrian Academy of Sciences, AUSTRIA

## Abstract

Iberian late prehistoric stelae stand out as a significant expression of European prehistoric art. For well over a hundred years, the context of use of the c. 300 Iberian stelae known to this date has been intensely discussed. This debate, however, was inherently flawed and limited in its scope, as almost all the available examples were found by chance, and no good-quality empirical evidence was available to understand their primary context. In this paper, we present the first conclusive scientific evidence demonstrating that Iberian late prehistoric stelae were used both as grave markers and landscape landmarks associated with pathways. This incontrovertible evidence stems from fieldwork undertaken in June 2022 and September 2023 at the site of Las Capellanías (Cañaveral de León, Huelva) in south-west Spain, including field walking, geophysics and excavation. The ground-breaking discoveries made at this remarkable site within barely a year reveal the contextual association of three different stelae to as many graves within the context of a large long-standing burial complex. Specifically, in this paper we study Stela #2 through a broad combination of methods ranging from geoarchaeology to luminescence dating and archaeoastronomy, as well as multiple digital visualization and remote sensing techniques. This multidisciplinary approach provides data that reveal multiple lines of evidence concerning the context and temporality of the stela, its manufacture and its graphic design. Altogether, this lays out a much needed and long-awaited reliable empirical base to understand where, when and how Iberian prehistoric stelae were used.

“We hewed logs and built his pyre upon the tallest headland where it runs out above the sea: duly we made his funeral, bewailing him with bitter tears. After body and armour were quite burned away, we piled a mound over them and to crown it dragged up a monolith”, Homer, *The Odyssey* (London, Arcturus, 2021. Trans. by Alexander Pope). “As he spoke he began stripping the spoils from the son of Paeon, but Alexandrus husband of lovely Helen aimed an arrow at him, leaning against a pillar of the monument which men had raised to Ilus son of Dardanus, a ruler in days of old”, Homer, *The Illiad* (Project Gutenberg, 2022. Trans. by Samuel Butler).

## Introduction

Late prehistoric statuary variously described as menhirs, statue-menhirs or stelae is common in several regions of western Europe, notably France, Iberia, the Alps, Greece and the western Mediterranean Islands [1–8]. In Iberia, since the publication of the Solana de Cabañas (Cáceres, Spain) ‘sepulchral slab’ in the late 19^th^ century [9], prehistoric stelae have been a major topic of debate. A large body of literature, including six books and over two-hundred papers, bear witness to the significance of these monuments, dating from the 3^rd^ to the 1^st^ millennia BC, and of which almost three hundred examples are currently known – see [10,11] for the latest overall syntheses and an overview. A substantial part of the long-standing and still-ongoing debate around Iberian late prehistoric stelae has focused on their context. This is particularly the case with the subgroups characterized by personages wearing a special, crescent-like ‘headdress’ (sometimes referred to as ‘diademadas’ – or ‘diademated stelae’) or surrounded by a panoply of weapons (called ‘warrior’ stelae). These two groups of stelae have a similar geographic distribution along the middle basins of the main rivers draining into the Atlantic across western Iberia, the Guadalquivir, the Guadiana, the Tagus, and the Duero. For a long time, they were also seen as symbolically and socio-culturally apart, a notion that recent discoveries in Almadén de la Plata and Cañaveral de León, in south-west Spain, have dispelled (see [8,10], for recent syntheses).

In this paper, we present the first conclusive scientific evidence demonstrating that Iberian late prehistoric stelae, and particularly the so-called ‘headdress’ and ‘warrior’ series, were used as monuments, *both* in burial commemoration and in territorial and landscape marking with a strong association with pathways. This evidence stems from fieldwork undertaken at the site of Las Capellanías (Cañaveral de León, Huelva) in south-west Spain, where in 2019 one such stela was found by chance [12], including field walking, geophysics and two excavation campaigns (carried out in June 2022 and September 2023). This investigation led to the discovery of a large burial complex composed of several cairns and cist-like chambers, as well as a second and third stelae.

Throughout the 20^th^ century, the problem of defining the context of use of Iberian warrior stelae remained unresolved. The steady discovery of new stelae over the years did not contribute to clarify the problem of their context, as, in most cases, new stelae were not found as a result of archaeological fieldwork, but as chance-finds in the course of agricultural work (ploughing, terracing, etc.). In addition, most stelae did not appear to be associated to immediately visible or recognisable archaeological features such as material culture or architectural remains. The alleged presence (almost always through unverified reports) of ashes or burnt (possibly) human bones, urns, and occasional (and tenuously supported) connection to funerary architecture (pits, cists, mounds), suggested an association with burial practices for a number of stelae. That was certainly the case of Solana de Cabañas (which was already referred to as ‘sepulchral’ by Roso de Luna), but also a group from Hernán Pérez, El Cerezal 1 (including La Corra and La Coronita, both missing nowadays) in Cáceres, Granja de Céspedes in Badajoz, Ervidel 2 in Beja, Ribera Alta/Córdoba 2, Cortijo de la Reina 1 and 2 and Cerro Muriano 2 in Córdoba, Setefilla in Seville or Haza de Trillo-Toya in Jaén [9–22]. Due to the lack of verified contextual evidence (to burial features or otherwise), in the 1990s, the role of warrior stelae as territorial markers, in association with pathways, mountain passes, fords, and other geographically-significant loci, was highlighted [23,24].

Two small excavations undertaken in stelae find-spots did not yield positive results [20,25]. In addition, the only ‘warrior’ stelae with recognisable contexts properly documented through archaeological methods (those of Setefilla, Cancho Roano, Pocito Chico, and Arroyo Manzanas) appeared to have been found in a secondary position resulting from a reuse, which prevented a straightforward understanding of their primary context of use [10]. The Cancho Roano stela was found re-used as a doorstep at the main entrance of a late Iron-Age building, defined variously as a ‘palace’ or ‘sanctuary’ [20,26]. Pocito Chico (Cádiz) is a fragment of a stela that was found re-used as part of the fabric of the wall of what the excavators described as a ‘hut’. According to a radiocarbon date from a non-articulated animal bone and the assemblage found as part of the infill, this hut was presumed to have been decommissioned by the 8^th^ century BCE [10,27,28]. The Setefilla stela was found covering a grave containing various remains of uncertain age, most probably belonging to the early and late Iron Age [13,29]. Finally, Arroyo Manzanas was found reused as part of the floor of a late Iron Age house [30].

Thus, suggestions were made that almost all Iberian stelae were inherently “decontextualized” [20,31–33] or that, perhaps, they possessed a certain ‘landscape setting’ but lacking direct architectural context [24]. Departing from “observational deficiencies” derived from the fact that most stelae had been found by chance, their “decontextualisation” became accepted by default [34]. As a result, as new stelae were discovered, almost always by chance, their iconographic details were meticulously recorded and scrutinized, but the context and setting of their find spots were largely ignored: no geophysical survey, intensive field walking or excavation of the stelae find spots (except for the two mentioned above) were ever carried out. Such epistemological approach to these important monuments would not experience any significant changes until the late 2000s and 2010s, when high-resolution fieldwork was conducted within the find spots of some newly discovered stelae with promising, but not fully conclusive results [35–40].

**Fig 1:**
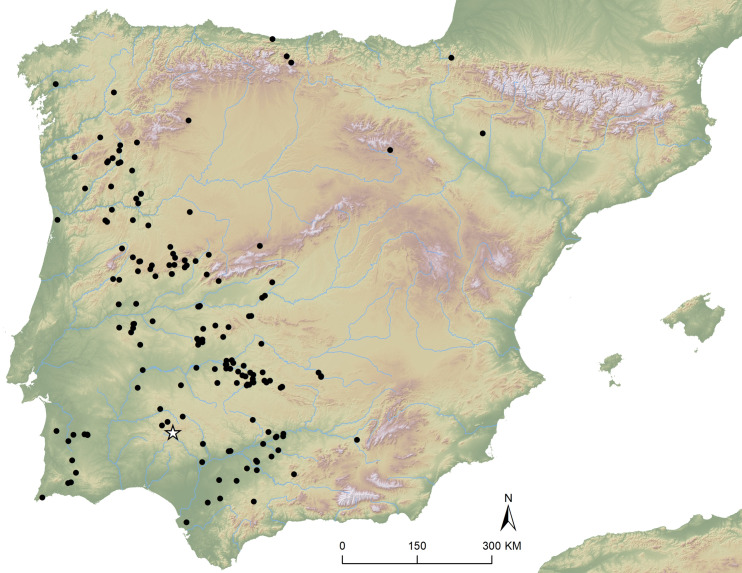
Geographic distribution of Iberian Late Prehistoric stelae. Printed under a CC BY 4.0 with permission from Marta Díaz-Guardamino.

**Fig 2:**
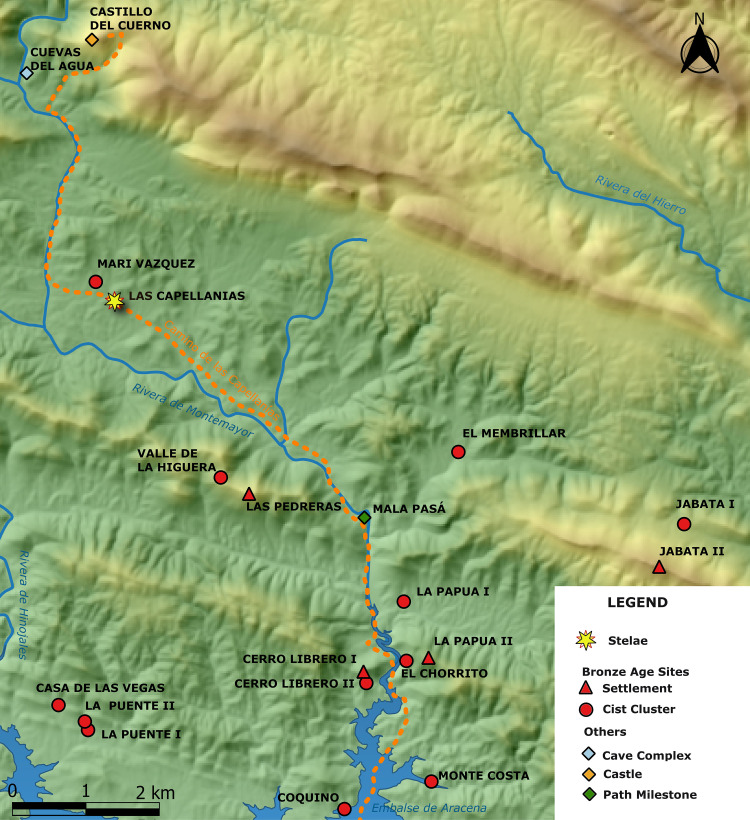
Location of Las Capellanías and nearby sites. Printed under a CC BY 4.0 with permission from Timoteo Rivera-Jiménez.

In this article, we present the Cañaveral de León #2 stela, the first Iberian warrior stela to have been found in a stratified position within its potentially primary context of use (a necropolis) and documented during archaeological excavations. Stela #2 was discovered within a burial mound during scientific excavations carried out in June 2022 at the site of Las Capellanías, where a first stela (designated as Stela #1) had been found by chance in 2018 [12] (Figs 1, 2, and 4), and a third (to be described in a future publication) was found as recently as September 2023. The discovery of these three newly found stelae in association with a major burial complex sheds completely new light onto the context, use, character and temporality of Iberian prehistoric stelae.

**Fig 3:**
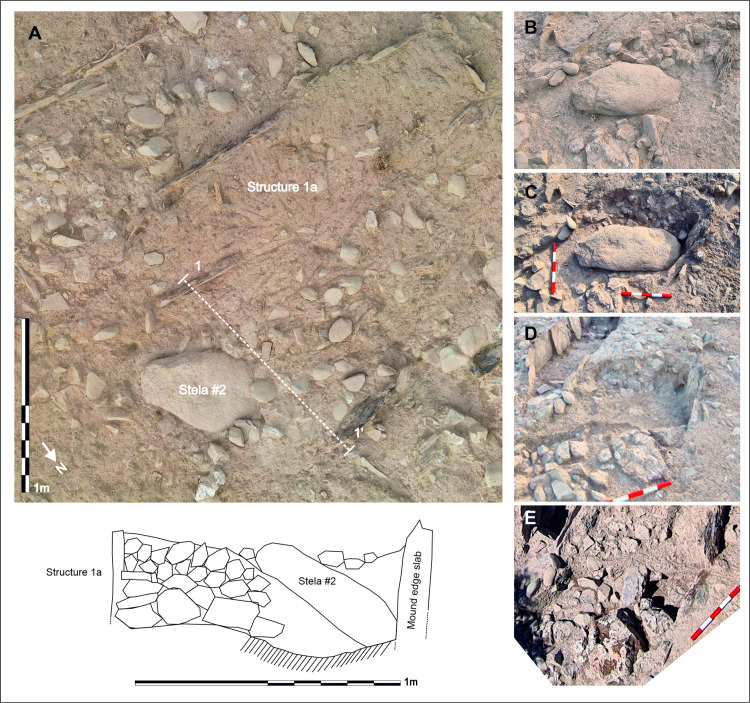
Excavation process of Stela #2 at Las Capellanías burial complex. A) Orthoimage of the stela and surrounding structures upon discovery; B) and C) The stela in the process of being excavated and ready for lifting; D) Soil deposit beneath the stela; E) Structural elements of the mound after removal of the stela and excavation of soil deposits. Bottom left: Section of the stela context located between Structure 1a and the slabs outlining the peristaltic ring of the mound. Orthoimage: David Wheatley. Photographs, annotation and section: Marta Díaz-Guardamino.

## The stela

**Fig 4:**
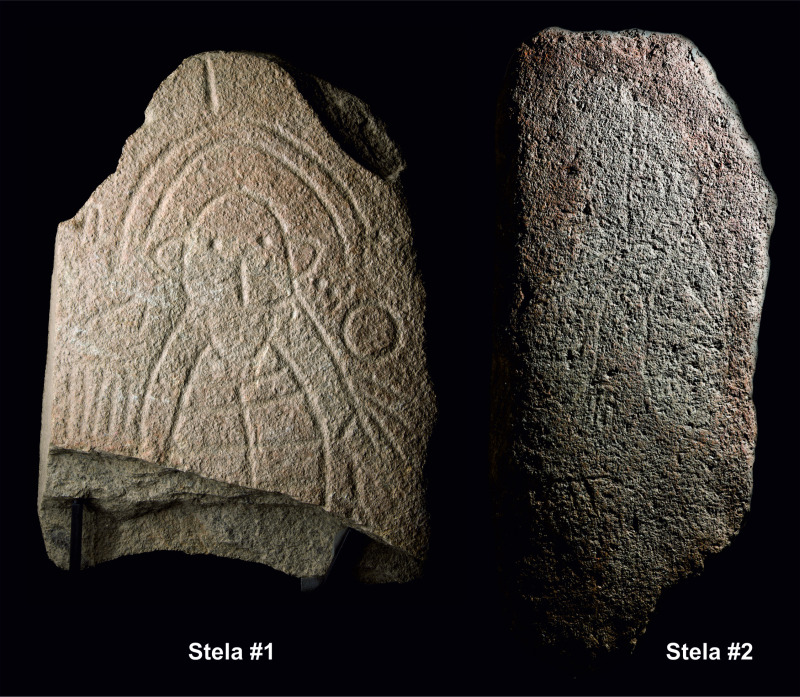
Photographs of stelae #1 and #2, once cleaned. Stela #1, is missing the bottom part and measures 0.95 m high, while stela #2 is complete and is 1.23 m high. Photographs: Marta Díaz-Guardamino.

The Cañaveral de León #2 stela was first discovered on Saturday June 4^th^ 2022 as the walls flanking the Las Capellanías pathway, cutting through the site, and the shrubs and top soil around it were removed by means of a machine in order to clear the way for the excavation work, which started on Monday June 6th. The stela was facing down and therefore its graphic motifs were not visible (Fig 3A). However, its geology, size, shape and dressed surface strongly suggested its sculptural nature right from the start. It remained *in situ* through most of the excavation campaign until June 21^st^, when it was lifted and its character as a stela confirmed (see Figs 3C-D; 4). This took place at night, as soil samples for OSL dating were retrieved from the layer of soil found directly beneath the stela. The stela was then brought to the Cañaveral de León City Council, where it was kept until July 20^th^, after which it was transported to the Huelva Archaeological Museum, where it is currently kept. It is worth noting its good state of preservation, as no significant portion of it appears to be missing. In addition, the stela does not seem to be intensively weathered, which suggests that it was not in an open-air context (exposed to weathering by wind and rain), for a long period of time. No traces of lichens were found on its surface either. It has a total height of 1.23 m, with a width of 0.6 m and a thickness of 20 cm. Its estimated weight is 112.5 kg (Fig 4: Stela #2).

### Geological characterization

A small sample was taken from the back of the stone to carry out thin-section petrographic analysis. A Nikon Eclipse LV-100 POL petrographic microscope connected to a Nikon DS-Fi1 camera, with a 5.24 mega-pixel 2/3-inch sensor was used to obtain the images of the thin section. The camera was connected to an Intel Pentium 4 2.66 GHZ with the NIS-Elements image capture software.

**Fig 5:**
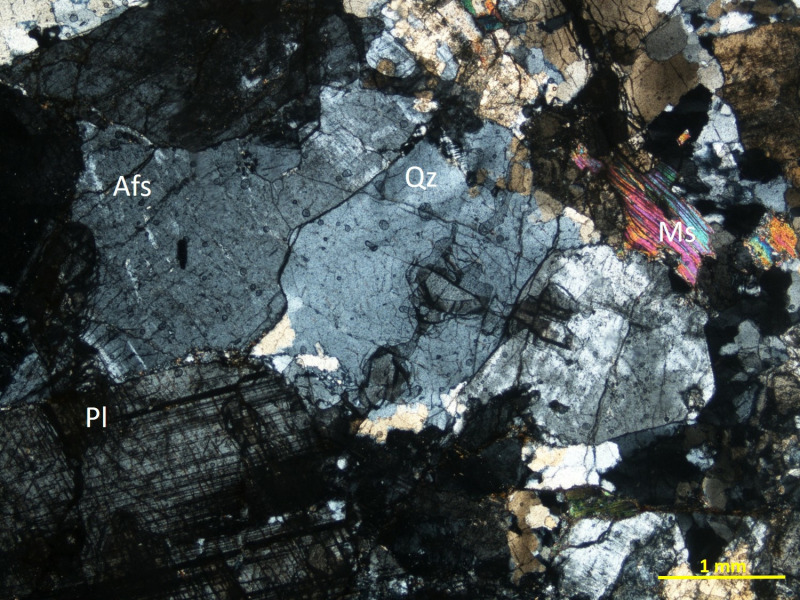
Photomicrograph of the thin section of the stela. Minerals: Afs, K‐feldspar; Ms, muscovite; Pl, plagioclase and Qz, quartz. Printed under a CC BY 4.0 with permission from Teodosio Donaire Romero.

That sample revealed that the stela was sculpted from a remarkably homogeneous, slightly deformed, fine to medium-grained muscovite-bearing leucogranite showing locally miarolitic textures (this is a type of granite, the term which is used in the remainder of the article for conciseness). It shows a massive structure and an isotropic fabric. It has an hypidiomorphic, inequigranular texture and the main primary constituents are K-feldspar, quartz, plagioclase (albite) and minor muscovite and biotite (Fig 5). Alkaline feldspar occasionally shows microcline twinning and perthitic textures. Although these rocks share many petrographic features with neoproterozoic leucogranites described in the Olivenza-Monesterio antiform [41], about 25 km northeast of Las Capellanías, there is no certainty as to the exact provenance of the rock used to manufacture Stela #2 of Cañaveral de León, which may, potentially, come from more distant sources.

While different from the foliated rhyolite porphyry of Stela #1, found barely 12 m to the south, the granite of stela #2 is not a rare occurrence among Iberian ‘warrior’ stelae. In total, about 70 out of 150 examples for which reliable lithological characterisations are available, were made of the same material. Most of them occur in northern-western-central Iberia (southern Galicia and north Portugal, the Portuguese Beiras, and the Spanish provinces of Salamanca and Cáceres), although some are also known in the south, including Cancho Roano and Quintana de la Serena (Badajoz, c. 130 km to the northeast of Las Capellanías), very close to each other, and Burguillos (Seville, c. 80 km to the southeast of Las Capellanías).

### Slab and rock art *chaîne opératoire*

**Fig 6:**
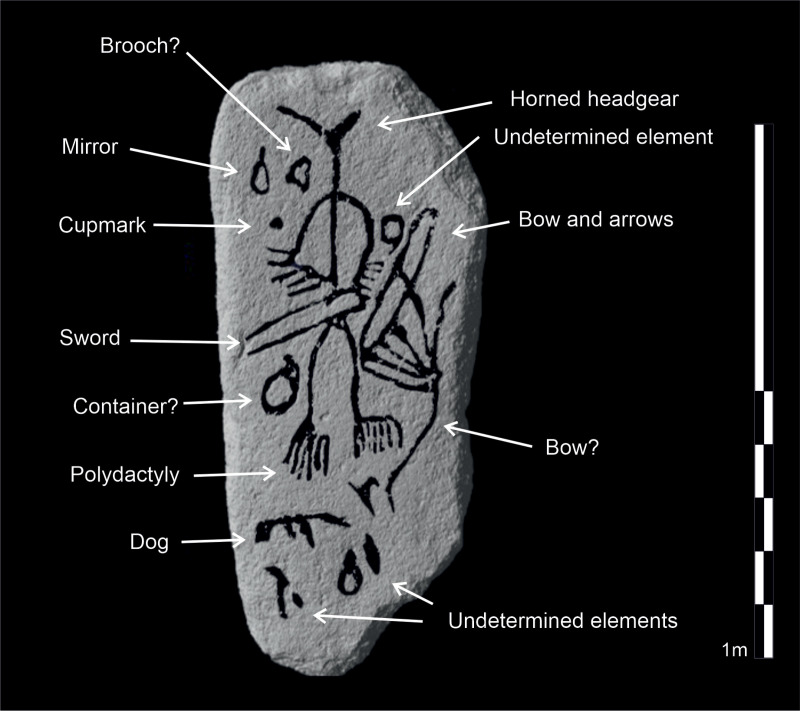
Synthesis and interpretation of the motifs depicted on the stela. Design: Marta Díaz-Guardamino.

**Fig 7:**
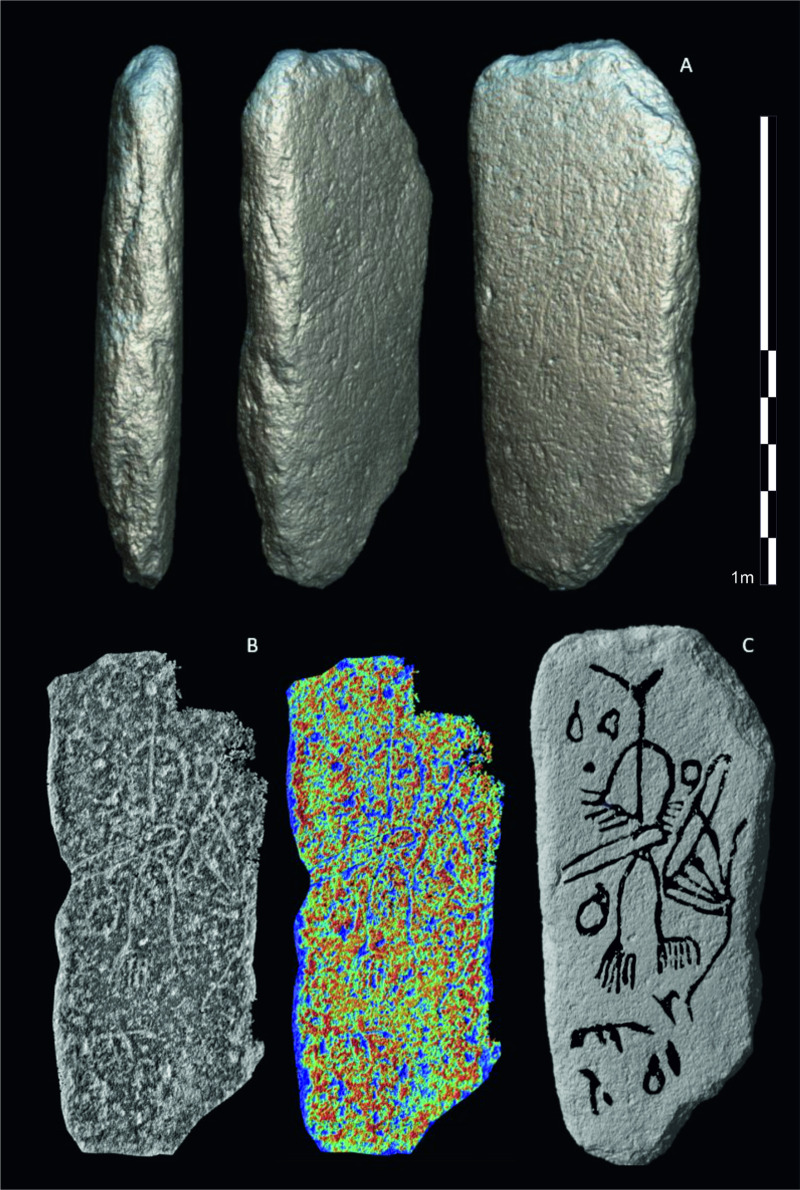
A) 3D renderings of the slab, on all its sides; B) Enhanced visualizations of the carved area through the application of TVT (https://tvt.dh.gu.se/); C) Interpretation of the carvings generated through interactive semi-automatic colorization. Design: Marta Díaz-Guardamino.

For the study of the stela morphology, and the technology and iconography of its carved motifs (Fig 6), close-range, HD SfM photogrammetry and Reflectance Transformation Imaging (RTI) were applied, while Digital Image Analysis (DIA) was used for pigment detection [42,43]. For the creation of a 3D model, 197, 24.5 MB photographs were processed in Agisoft Metashape using the High-Quality settings. The resulting 3D model has 1 mm accuracy. The control scale bars’ total error is 0.757 mm. The mesh has more than 6 million vertices. PTM and RTI files were produced from 108 HD photographs, with the free software RTIBuilder (https://culturalheritageimaging.org/). Both techniques, used for the study of slab morphology, rock carving techniques and sequence of manufacture, and iconography, are described in various recent papers - e.g., [38]. For DIA [42,43] the images obtained were uncorrelated by means of Principal Components Analysis (PCA) using the HyperCube software package (Robert Pazak, USACE, Alexandria, VA, USA) in order to perform digital image analysis (DIA) following the protocols explained elsewhere [42,43] and already implemented in previous work by the same team [12,36,38,39]. SfM photogrammetry produced a HD 3D reconstruction of the stela that shows the key morphological characteristics of the slab and the markings on its surface (Figs 6 and 7). A medium resolution version of the 3D model can be explored here: https://skfb.ly/oAPzq, and a high resolution 3D model can be downloaded here: https://doi.org/10.15128/r2sb397830v.

The stela is a roughly rectangular-shaped slab, with preserved original sides which are slightly irregular on the right and upper-right, although these irregularities seem to be related to the original morphology of the extracted/collected granite block (Fig 7:A, see also 3D model: https://skfb.ly/oAPzq). The reverse (i.e., plain side) of the stela has a few irregularities with marks of rough levelling, while the obverse, where the rock art carvings are found, is flat and smooth as a result of intensive levelling and grinding work performed before the carved decoration was made (Fig 7:A).

**Fig 8:**
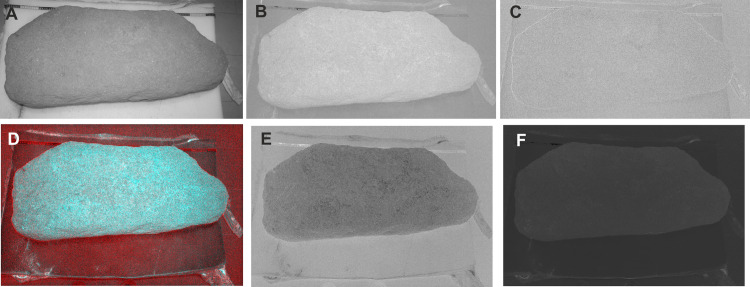
Several results of DIA applied to image 6909. A) Band obtained by PC1; B) Band obtained by PC2; C) Band obtained by PC3; D) False colour image (combination 322); E) Results of the subtraction of PC2 band to PC3 band; F) Results of Ferric Pigments Index. Design: Miguel Ángel Rogerio-Candelera.

To test whether the intensive surface preparation work had been complemented with colour treatment, as it was found on stela #1 [12], DIA was used. The original images showed a high correlation among the different bands (Table 1), ranging from 92 to 98%. In cases like this, PCA is the best fitted technique due to its huge capabilities for uncorrelating highly correlated images. PCA of the studied images yielded very good results as the distribution of variance was typical of these kinds of highly correlated image datasets (Table 2), clustering about 97% around the axis of the first Principal Component and leaving the remaining less than 0,1% on the Third Principal Component. Typically, if the remains of painting do exist (even if they are difficult to visualize), they are graphically reflected in the band corresponding to the third Principal Component. No great difference among the three bands of the PCA can be appreciated (Fig 8A-C). The different combinations of bands employed to produce false colour images show a great homogeneity on the surface, not allowing the detection of elements that could be interpreted as intentionally painted (Fig 8:D). Only some black pixels can be appreciated when extracting the pixel values of PC2 band to PC1 band (Fig 8:E), but the implementation of a Ferric Pigments Index (FPI) [44] is clearly negative (Fig 8:F). Altogether, this evidence shows that colour treatment was not applied to the Cañaveral de León #2 stela.

**Table 1:** Band correlation of images of Cañaveral de León stela #2.

Image id.		PC1	PC2	PC3
	**PC1**	1	0,98373681	0,92881127
6905	**PC2**	0,98373681	1	0,97600379
	**PC3**	0,92881127	0,97600379	1
	**PC1**	1	0,98476849	0,92994222
6909	**PC2**	0,98476849	1	0,97503699
	**PC3**	0,92994222	0,97503699	1

**Table 2:** Variance explained by the Principal Components.

Image id.	Principal Component	Eigenvalue	Explained variance (%)
	1	4798,272	97,57531560
6909	2	114,678	2,33203579
	3	4,556	0,09264859
			[Ʃ=100]
	1	4914,351	97,53217950
6905	2	119,970	2,38097270
	3	4,376	0,08684785
			[Ʃ=100]

On the smooth, levelled obverse side of the slab, there are a series of carved motifs, most of which are typically found on warrior stelae (Fig 7B-C). On the centre of the stone ‘canvas’ there is a rather large schematic human figure with curved extremities ending in hands and feet with large, clearly outlined fingers and toes. Something quite remarkable is that the warrior depicted on this stela shows post-axial polydactyly in both of their feet, that is, that each foot has six toes. The digits of hands and feet are, typically, carefully (and numerically correctly) represented on Iberian stelae, so this is an intentional and significant physical trait, with implications which are discussed below. The panoply of weapons most immediately associated with the figure includes a sword set across the waist (with the tip pointing to the right of the warrior), as well as ‘horned’ headgear. To the left of the human figure, by the shoulder, two further motifs can be seen, perhaps the schematic depiction of a mirror and a brooch. Below the hand and sword, on the left side of the figure, there is a round element, which could perhaps represent a container, but this is quite uncertain. To the right of the individual, in the upper part, there is a square-shaped element which is difficult to identify, and a bow fitted with what appear to be various arrows, as well as an elongated element that is difficult to interpret—perhaps a second bow? In the lower section of the obverse there are further elements, including a possible quadruped, which could be a dog (although horses are also found in some ‘warrior’ stelae, in association with two-wheeled chariots) and possible remains of carvings that have been partially effaced. As mentioned above, no carved motifs were identified on the back or sides of the slab.

**Fig 9:**
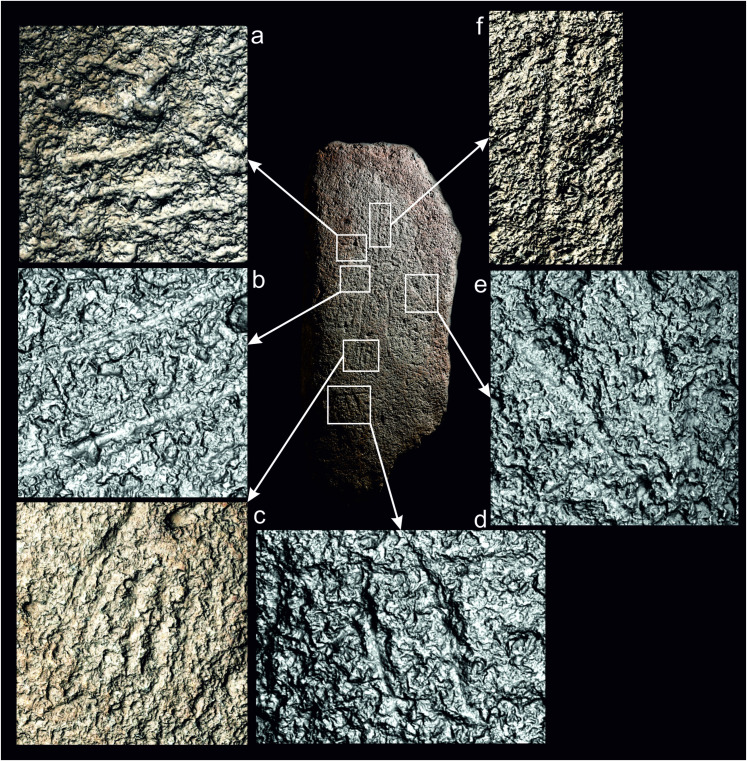
RTI visualizations of the texture of grooves outlining: a) fingers; b) sword outline; c) foot toes; d) quadruped; e) arrows; f) torso. Design: Marta Díaz-Guardamino.

Detailed RTI surface examination showed that most of the grooves were finished through abrasion (Fig 9b, c, e - the full RTI and PTM files of the stela can be downloaded from here: https://doi.org/10.15128/r2k643b125t). This is the case of all the motifs examined except for some linear sections of the human body, where the surface is rougher, and the granular texture of the granite slightly stands out (Fig 9:f). This may be due to the relatively low-quality work of the carvings, as the preservation of the stela surface is excellent and the probability that this rougher groove surface was produced through erosion is low. Pecking marks in these areas are difficult to identify on account of the coarse texture of the granite. Some grooves show marks of incisions that were made after the groove was smoothened (Fig 9a, d). These incisions could have been made during the initial manufacturing process or at a later stage to revive the carvings.

The sequence of carving of the different motifs is difficult to discern, as there are only a couple of superimpositions. The most plausible sequence would start with the carving of the human figure, the largest graphic element, which also presides over the composition, occupying the centre of the slab. This was carved simultaneously with the belted sword, as the carving of the body/legs complements that of the waist, and the horned headgear. Then, all the remaining elements would be carved around the human figure (Fig 6).

**Fig 10:**
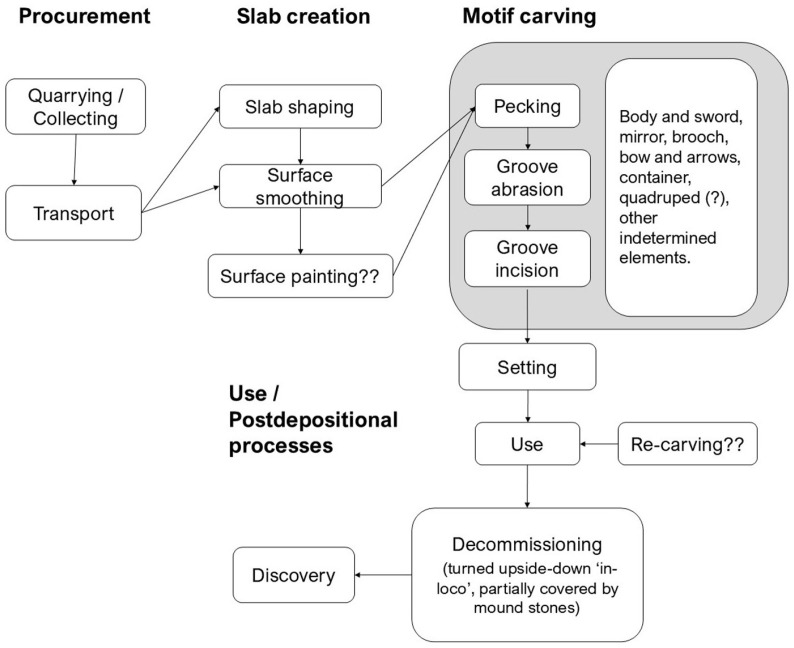
*Chaîne opératoire* of manufacture and biography of the stela. Design: Marta Díaz-Guardamino.

Overall, the *chaîne opératoire* of manufacture of stela #2 (Fig 10) shares some traits with those documented on other stelae whose technological characterization has been appraised recently [45]. In all cases significant time and effort was invested on the preparation of a flat and smooth ‘canvas’ where the motifs were to be carved. However, the specific techniques employed to carve motifs and their sequence of manufacture are rather different from one case to another. This could be related to the characteristics of the lithology of the stone the stela is made of, the structure of the individual boulder, and/or the type of tools employed for carving [45]. When comparing the manufacture of stela #1 of Cañaveral de León [12] with that of stela #2, it is apparent that the carving work of the former was more carefully executed, with a better-quality finish, while the carvings of stela #2 seem to have been made in haste, possibly without previous planning, as an *ad hoc* execution.

## Context and dating

**Fig 11:**
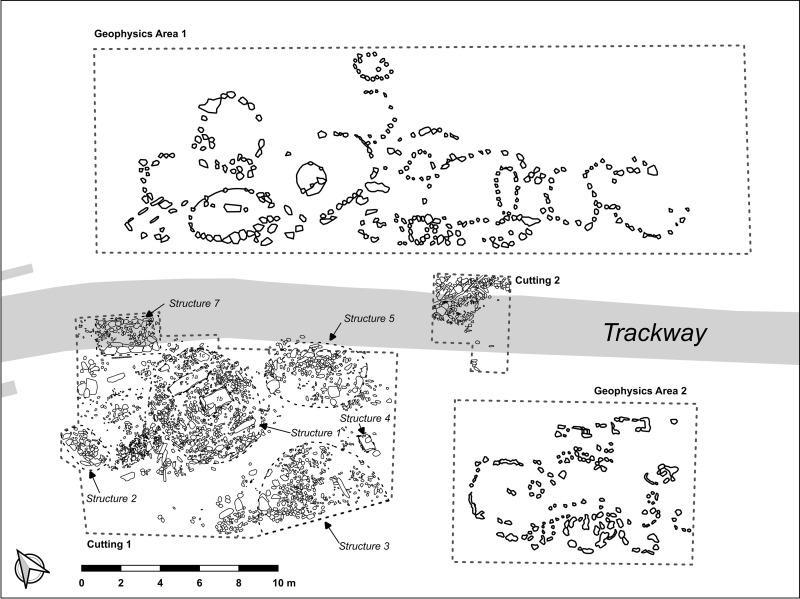
Synthetic plan of the Las Capellanías burial complex after the June 2022 excavations. Printed under a CC BY 4.0 with permission from David Wheatley.

The excavation undertaken in June 2022 revealed a large funerary complex, partly overlaid by the Las Capellanías pathway, exactly on the same spot where stela #1 had been found [12]. Indeed, some of the stones visible on the surface of the pathway, which were initially interpreted as a pavement, were soon discovered to be part of funerary structures. The excavation work and geophysics show that this necropolis extends over an imaginary square with sides approximately (at least) 60–50 m in length (Fig 11), although it seems to expand further to the south, towards the Rivera de Montemayor river, as suggested by the dense scatters of stone materials, particularly white or milky quartz, on the surface. In total, after the excavation work undertaken in June 2022 and September 2023, more than 20 structures have been identified based on the available data (Fig 11).

In addition to exposing the layout and organization of the burial complex, the 2022 fieldwork season was primarily focused on the delimitation and excavation of Structure 1, where stela #2 was found (Fig 3:A; See S1 Fig). Structure 1 is a 6.5 m-wide circular cairn made with various types of rock, including milky quartz, schist and greenish pebbles of volcanic rock (mostly ophite) and delimited on its perimeter by large slate slabs deeply stuck into the ground. Inside Structure 1 two cists were discovered, which are referred to as structures 1a and 1b respectively.

Structure 1a is located on the northern half of the cairn, at a distance of around 1m from its edge (marked by the vertical slabs that support it). It is a rectangular cist delimited by 10 vertical slate slabs (two appear to be missing on its norther side) of a highly unusual and striking purple colour. This cist has the following measurements: 2.31 m in length, 36 cm in maximum width and 46 cm in maximum depth (Fig 3:A). Based on its size, Structure 1a is significantly larger than most Early/Middle Bronze Age cists, which are commonly found in the Iberian south-west. These cists, dated by approximately 30 radiocarbon dates on human bone to c. 2200–1500 BC (with just one slightly later exception from Pessegueiro), usually measure about 1.5 m in length and 0.5 m in width [45–49]. Originally, Structure 1a must have been covered by a series of horizontally-laid slabs, now missing, as well as the stones of the cairn, all of which were probably re-used to build the flanking walls of the Las Capellanías pathway in medieval or modern times. In general, it seems likely that the tumular mass of Structure 1 has lost between 20 and 50 cm of height on its central part, on account of such reuses.

The excavation of Structure 1a revealed that it was almost completely empty. On the upper part of its infill, centred within the cist, an accumulation of stones was found, including broken portions of slate slabs, nodules of milky quartz and pebbles of volcanic rock, all of which amounted to 109.9 kg of stone. Given its concentration and density, this accumulation of stones could be deliberate, rather than simply resulting from a collapse. Other than the stones, half a dozen tiny fragments of hand-thrown pottery and some minute remnants of charred material were identified, all of which were recorded and are currently being studied. No human remains were identified. The absence of bone is a recurrent problem in most prehistoric tombs in the region and is usually explained by high soil acidity [46]. According to the publicly accessible information provided by the Andalusian regional government (available at http://www.juntadeandalucia.es/medioambiente/mapwms/REDIAM_propiedades_quimicas_suelo_biomasa_forestal) Las Capellanías soils are only slightly acidic (6–6.5), which leaves the issue of bone preservation open for now. Furthermore, the absolute absence of material culture remains in Structure 1a suggests a thorough and systematic ‘emptying’ of the tomb. This points to a careful and systematic removal of its contents that goes beyond what is usually found in cists ‘looted’ in recent times.

Structure 1b, located 85 cm to the south of Structure 1a, occupies a more ‘central’ position inside the cairn and presents measurements much more in line with ‘standard’ Early/Middle Bronze Age cists of the Iberian south-west. This structure has been excavated in September 2023 and is still under study.

Barely 30 cm to the NE of Structure 1a was stela #2, facing down and with a slight inclination of 20° (Fig 3). The (decorated) obverse was facing down, with the upper tip of the stela slightly below the base of one of the vertical slabs delimiting the cairn (Fig 3C-D, Section). It is unclear whether that can be explained because the vertical slab was placed after the stela (which could mean that the stela was positioned where it was found when the cairn was made or sometime after the cairn was made as a result of a possible ulterior refurbishment) or whether this is because the vertical slab has tilted towards the south over time, thus ending up leaning slightly over the stela. The middle part and upper tip of the stela were covered by the same type of stone and soil material forming the mound of Structure 1. Once the stela was lifted, it became immediately obvious that the stone-and-soil infill of the cairn was also under most of it (Fig 3D-E, Section), which suggests that the stela had been deliberately buried within the mound. OSL samples were taken from the soil sediments found underneath the stela (Fig 3:D) when the stela was lifted at night. A trowel was used to scrape the surface of the sediment layer underneath the stela, retrieving soil from up to c. 2 cm depth and transferring it to two black 50mm PVC tubes that were subsequently sealed.

The soil layer found underneath the stela reached its upper tip (to the North) (Fig 3:D), where it was covering a series of large stones appearing to delimit a small cavity with an infill of loose soil (Fig 3:E, Section). This cavity had an approximately oval shape measuring 50 cm in a N-S direction, and 43 cm in an E-W direction, with a depth of around 30 cm. A section was cut through this feature, revealing an ashy and loose infill, with small fragments of slate. A minute (less than a square cm) fragment of pottery, apparently burnt, was found in it. Additional samples from the infill of this feature were taken for OSL dating. This feature could perhaps be interpreted as a socket in which the stela had stood prior to its burial within the mound. Indirect support for this hypothesis comes from the fact that immediately to the north-east, and adjacent to it, a very large block of milky quartz, about 50 cm across, was found. This large block of quartz was found next to the possible socket feature, halfway across the stela. The excavations undertaken in 2022 and 2023 have revealed, beyond any doubt, a strongly patterned use (and associated symbolic significance) of milky quartz blocks at Las Capellanías, which are otherwise used in many prehistoric burial features across the region [50] and indeed have been found in connection with another ‘warrior’ stela at a nearby location, in the municipality of Almadén de la Plata [35]. The large block of milky quartz placed next to where stela #2 was found is by far the largest of such blocks at Las Capellanías. Both its position and size suggest that it marks a symbolically charged spot within the structure. Two Optically Stimulated Luminescence (OSL) ages were obtained from the soil samples retrieved from underneath the stela (Table 3). The measurements were performed with aliquots, each containing c. 2k grains. The dose rates were modelled and corrected using measured water content, grain size and etch attenuation factors (for details, see dose rate determination sub-section in S2 File). The reported ages are given in years (ka) (for reference the measurement year is 2024). Both OSL dates are consistent, with a 1σ age range of 2340 and 1680 BC, a period of time otherwise usually referred to as Early Bronze Age (c. 2200–1600 BCE) (Table 3) in southern Iberia. While these ages are consistent with the chronology of burial mounds like Structure 1 found throughout the Iberian south-west (see discussion below), they are older than the chronology usually attributed to ‘warrior’ stelae, which scholarship spanning one hundred years has set between c. 1425/1300–850/750 BC (Late Bronze Age and the beginning of the Early Iron Age) [see 28 for a summary]. This raises a number of important questions, which are discussed below. Before addressing these questions, there is an important caveat to note. We cannot rule out that the sediment collected could have contained (quartz) grains with different histories of optical resetting before burial. While the grains within the uppermost surface of the sediment (< 1 cm) are expected to have been repeatedly exposed to daylight until the stela was placed over it, the grains at a deeper level are likely to have remained in dark conditions, unless disturbed. The deeper grains would yield OSL ages related to their deposition and burial, in this case the formation of the mound. The mixing of grains with significantly different optical resetting histories has an averaging effect in the resulting OSL age.

**Table 3:** Burial doses (D_b_), Dose rates and Ages for the analysed samples.

Sample	Lab ID	Environmental Ddose rate (Gy/ka)	D_b_ (Gy)	Age (ka)
LC22 UE1C OSL0001	DLL24 S.179	3.32 ± 0.24	13.08 ± 0.46	3.94 ± 0.32
LC22 UE1C OSL0002	DLL24 S.180	3.32 ± 0.24	13.36 ± 0.50	4.03 ± 0.33

Archaeoastronomical measurements were taken to understand the orientation of stela #2 and the two associated burials inside Structure 1 (Table 4). The azimuth data were obtained with a professional compass (SUUNTO 360-R) and therefore have been corrected for magnetic declination effects. Such measurements have an accuracy of ¼º. The horizon height measurements were obtained with a professional clinometer of the same brand, with 1/3º accuracy. In addition, a series of 16 photographs were taken to make a cylindrical panoramic view. The photos were taken from nearby the location of Structure 1, and the final model is obtained with the Hugin software. This model is cleaned of sky and included in the Stellarium software (version 1.23.2) for its sky simulation.

**Table 4:** Orientation data for Structure 1.

Structure	Azimuth	Altitude of the horizon	Astronomical declination
1a	96.75	1	−4.9
1b	100	1	−7.5
1c (stela)	142.1	2	−37.1

The columns indicate the name of the structure, the azimuth in degrees, the altitude of the horizon in that direction and the astronomical declination corresponding to that direction in the horizon. All data is given in degrees (°)

**Fig 12:**
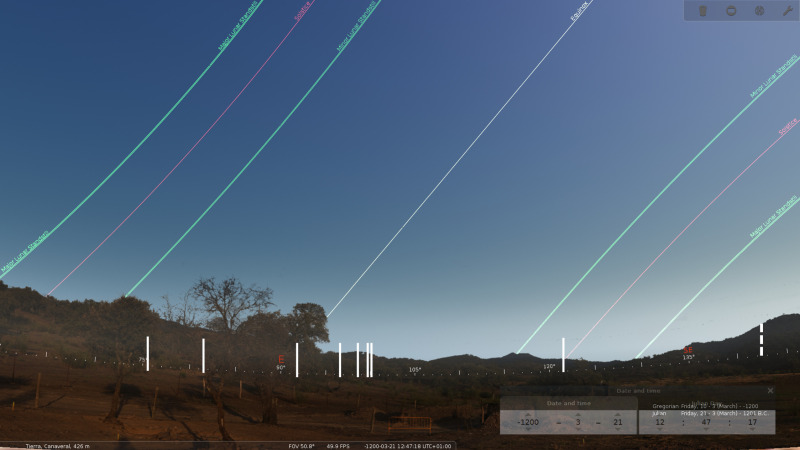
Panoramic view of the area of the necropolis towards the east, included in the Stellarium software. The red lines above the horizon indicate the movement of the sun in the summer (left) and winter (right) solstices. The white line indicates the path of the sun at the equinoxes. The green lines indicate the path of the moon at the lunastices. The solid white strokes indicate the orientation of the eight tombs, while the dashed stroke marks the orientation of the feature initially thought to be the socket of stela #2. Design: A. César González-García.

It is interesting to note that all declination values at Las Capellanías are between 24° and −24°, which corresponds to the extreme positions of the sun at the solstices for the epoch, but not that of stela #2 (Structure 1c) (Fig 12). The orientation of the possible socket feature found underneath the upper tip of stela #2 is more than 40° away from that of the two tombs located in Structure 1. The values for all the tombs in the necropolis (including structures 1a and 1b) are consistent with sunrise at different moments along the year, with an interesting prevalence of times during autumn/winter. In fact, Structures 1a and 1b do have quite similar orientations. In this sense it is interesting to note that the orientation of the potential pit documented beneath the upper tip of stela #2(Structure 1c) is at odds with this general pattern followed in the necropolis, and that the sun would not be visible in this direction in the horizon. It is also relevant that this orientation would not be consistent with any position of the moon in the horizon. The corresponding declination (−37.1°) would neither be consistent with the rising of any bright star at the time of use of the necropolis (assumed to span at least one and a half thousand years, between the late 3^rd^ and mid-1^st^ millennia BC). In view of these results, it is possible to conclude that while there are clear indications that the Las Capellanías tombs follow an orientation pattern possibly related to the rising of a heavenly body, most probably the sun, the orientation of the potential socket of the stela #2 does not seem to follow any astronomical relation, indicating that this feature was probably unrelated to the cists and the stela, as the OSL ages of the deposits underneath the stela (and covering this feature) suggest.

Altogether, combining all the contextual evidence gathered as part of this study, some relevant inferences can be made regarding the spatial position of stela #2: i) the stela was found as part of a burial mound (Structure 1), on whose northern limit it was embedded; ii) after perhaps standing up for an undetermined (but not very long) period of time, on this particular location or elsewhere in the necropolis, the stela was carefully deposited head down (and face down) as an intentional act of closure, termination or, perhaps, damnation. Given the lack of weathering and lichens on its surface, it is not possible to discard the idea that the stela was buried into the mound soon after its manufacture (or even immediately after); iii) the stela was intentionally buried close (c. 30 cm) to the NE edge of Structure 1a (large cist), adjacent to a large block of milky quartz, which is likely to have held a strong symbolic significance; iv) The two OSL age determinations obtained from soil samples lying directly beneath the stela albeit with limited precision (i.e., 2670–1350 BC ± 2σ or 2340–1680 BC ± 1σ,), indicate that its deposition was no earlier than c. 2700 BC (2σ) or 2340 (1σ).

## Discussion

The Cañaveral de León #2 stela is remarkable on account of both its context, graphic motifs and chronology. As noted in the introduction, the context and function of ‘warrior’ stelae have been hotly debated for decades. Up until now, only tenuous, doubtful and poorly understood evidence, or cases involving other types of stelae, connected them with funerary architectures and passageways. The best documented case is the stela of Alfarrobeira, located by the river Arade in the Portuguese region of the Algarve [51]. This stela is of the ‘Alentejan’ type, decorated with ‘anchor-like’ object, and conventionally attributed an Early/Middle Bronze Age chronology [10]). This stela was found in the 1950s laying by a small cist within an Early/Middle Bronze Age (EBA) funerary complex, Alfarrobeira 1, composed of a series of rectangular cairns containing one cist each. The excavation of the necropolis in the late 1980s revealed a socket on the north side of one of the most recent and peripheral cists (#2) where the stela would have been placed. Unfortunately, the stela was not stratified, and no human bone was documented in the whole necropolis (now covered by a dam), so the information available about the community buried there and the biography of the monument is very limited.

Another case is the warrior stela of Ervidel 2 (in the Lower Alentejo), found nearby two cists in Herdade do Pomar, one containing a female inhumation yielding a radiocarbon date within the Early Bronze Age (Table 5: ICEN-87) and two ceramic vessels, while the second had been looted [17]. Finally, it is worth noting the stela with ‘headdress’ of El Cerezal 1 (Cáceres), which oral reports indicate was found upright, next to a cist containing an urn, and also the ‘warrior’ stela and the stelae with ‘headdress’ of Hernán Perez 3–6 (Cáceres), which, again, oral testimony indicates that were found upright in an area where there were cists [16,18,22]. The necropolis of Herdade do Pomar is located in the middle of an extensive plain, close to an important copper mining area during late prehistory (Mina da Juliana), with no apparent spatial relation to natural pathways. Nonetheless, the sites of Cerezal 1 and Hernán Pérez are located within an important passage zone connecting the northern and southern halves of the Iberian Central Plateau through the Gata mountain range. Also interesting is the recently published stela of Alto de la Cruz de Piedra (Badajoz), found around 50 metres away from cists that are thought to date to the Bronze Age but have not yet been excavated [47].

**Table 5:** Radiocarbon dates for burial complexes mentioned in the text.

SITE	STRUCTURE	DATED MATERIAL	DATE	DATE BP	DATE BC 2σ	REFERENCE
Atalaia	Pit 33, Burial Complex II	CM	KN-I-200	4240 ± 50	3020-2620	Schubart, 1975
La Traviesa	Cist 20	CM	RCD-2110	3520 ± 95	2150-1650	García Sanjuán, 1998
Herdade do Pomar	Cist 1	HB	ICEN-87	3510±45	1954-1693	Barceló, 1991
La Traviesa	Cist 20	CM	RCD-2111	3420 ± 60	1890-1520	García Sanjuán, 1998
Pessegueiro II	Grave 16	HB	ICEN-867	3270 ± 45	1690-1430	Tavares da Silva & Soares, 2009
Pessegueiro II	Grave 16	HB	ICEN-868	3030± 40	1408-1129	Tavares da Silva & Soares, 2009
Vale da Telha	Cist 10	CM	Beta-232901	2920± 40	1258-1004	Gomes, 2015
Atalaia	Outside Burial Complex V	CM	KN-I-204	2870 ± 40	1192-922	Schubart, 1975
Atalaia	Grave 7, Burial Complex IV	CM	KN-I-201	2770 ± 50	1020-810	Schubart, 1975
Nora Velha	Burial VIIIA	CM	ICEN-1102	2720 ± 50	976-800	Monge Soares and Martins, 2013
Vale da Telha	Cist 13 (reuse?)	CM	Beta-345366	2620 ± 30	827-772	Gomes, 2015
Nora Velha	Burial VIIIB	CM	ICEN-1103	2520 ± 90	827-408	Monge Soares and Martins, 2013

Dated Material: CM (Charred Material), HB (Human Bone).

There are also other cases of stelae informally reported to have been found in connection with cremation urns (e.g., Cortijo de la Reina, Cerro Muriano), but these have not been confirmed by archaeologists. An exception is the Setefilla stela, documented during archaeological fieldwork in the 1920s. This was found covering a grave with urns, cremations and an inhumation, within the so-called ‘Orientalizing’ necropolis [13,27]. In this context, this stela has been interpreted as having been reused due to the uncertainty of the dating of the funerary context, but the possibility remains that the stela could have been positioned there as part of its primary use (see below).

Burial complexes dating to the Bronze Age and Iron Age analogous to those of Alfarrobeira and Las Capellanías including cists or cairns with one or more cists inside delimited by stone rings or rectangular structures, are known across the Iberian South-west [46–48,52–54]. As well as Alfarrobeira, there are other examples at Atalaia [55,56], Fernão Vaz [52], Provença [57,58], Pessegueiro and Quiteira [58–61], Vale da Telha [62], La Traviesa [46], and Nora Velha [63]. In general, the radiocarbon chronology available for those necropolises is rather poor, with few dates mostly obtained from samples of charred material due to the dearth of human bone (Table 5). On the basis of the available radiocarbon dates and material culture, Atalaia, Alfarrobeira and La Traviesa are dated to the Early/Middle Bronze Age, whereas Provença, Corte Cabreira, Pessegueiro, Vale da Telha and Quiteira appear to date to the Middle/Late Bronze Age (LBA) and Atalaia and Nora Velha to the Late Bronze Age (LBA) and Iron Age (IA). However, there are serious doubts as to the fine detail of the temporality of these complexes, suggesting the possibility that they experienced persistent multi-period use [64]. At Atalaia, the only three dates available suggest a temporality between the early 3^rd^ and late 2^nd^-early 1^st^ millennia BC, although the earliest of those dates is widely accepted as contradictory with the material context of the necropolis [56]. At La Traviesa, two dates set the use of the burials in the first half of the 2^nd^ millennium BC, which is in keeping with the morphology of the cist graves found there. In Pessegueiro II, dates on human bone provided an age between the 17^th^ and 12^th^ centuries BC for two burials deposited in a cist. In Vale da Telha material culture and radiocarbon dates on charred material indicate possible use of the necropolis between the 15^th^ and 11^th^ centuries BC, with some possible (but uncertain) reuse during the 9^th^-8^th^ century. At Nora Velha, two dates obtained for charred material set the use of this necropolis between the 10^th^ and 5^th^ centuries BC.

The most geographically close analogue for Las Capellanías Structure 1 is La Traviesa, located some 50km to the East as the crow flies. Cist #5 at La Traviesa, measuring 3.2 m by 1.3 m (and therefore of much bigger size that the rest of the cists surrounding it), was placed on a topographically prominent location and covered by a cairn of stones 6.7 m across supported by a ring of vertical slabs [46], and therefore not very much unlike Structure 1 at Las Capellanías. However, in terms of architecture, complexes like Atalaia, Alfarrobeira, Vale da Telha, Provença or Nora Velha appear to be more similar to Las Capellanías than La Traviesa, insofar they include multiple cairn structures (at La Traviesa there is only one) attached to each other, sometimes forming groups or clusters. Comparable necropolises dated to the Early Iron Age, like Mealha Nova and Herdade do Pego, have yielded epigraphic stelae with so-called Southwestern script, one of the earliest examples of alphabetic script known in Western Europe (dated from c. 8^th^/6^th^ centuries BC depending on the authors) [65,66]. It is in the periphery of this type of necropolises too where a new type of funerary structure appears around the 5^th^ century BC, the so-called monuments in π, which are very similar to structure 7 at Las Capellanías, usually holding cremations in urns [52,67,68].

Las Capellanías not only offers architectures that are akin to the burials and necropolises described above, but also, in the September 2023 still-unpublished season, it yielded a substantial amount of cremated human remains (currently under study) confirming, indisputably, their funerary use. The broad range of similarities that Las Capellanías’ funerary structures share with those dated to the Bronze Age and the Iron Age, as well as the material culture unearthed in 2023 and the OSL dates for stela #2, embedded in the mound of Structure 1 reported here, suggest that the use of the site and the warrior stela in it spanned several centuries from the late 3^rd^ and through the 2^nd^ and first half of the 1^st^ millennia BC. The excavations carried out in September 2023, which have involved several other burial features, and subsequent post-excavation analyses will greatly contribute to produce a better understanding of the site in this and other respects.

Notwithstanding, the Cañaveral de León #2 stela is also noteworthy on account of its graphic configuration. Firstly, its carvings are very schematic and have a rather sloppy manufacture. This is somewhat surprising, as it does not match the effort invested in the procurement of the raw material, a leucogranite which was brought, probably, from some distance. Warrior stelae made of granite in that region are not abundant but there are two known cases, namely Fuente de Cantos (Badajoz), c. 44 km to the north-east and Burguillos (Seville), c. 80 km to the south-east. These two stelae also resemble the Cañaveral de León #2 stela in the way the human figure was depicted, including how the anthropomorph was placed as the central figure of the composition, and similar stylistic traits (e.g., curved arms) (synthetic images of these and other stelae mentioned in this discussion can be found in [10]. The similarity between the Cañaveral de León #2 stela and that of Fuente de Cantos is particularly stark. It is relevant to note that the latter includes the depiction of a chariot, an element whose knowledge in Iberia is attributed to the Late Bronze Age. As noted above, the Cañaveral de León #2 stela shares some technical traits with other ‘warrior’ stelae (e.g., flat ‘canvas’) but was, nonetheless, made with an idiosyncratic technical approach. This was perhaps the result of limited skill/experience in carving, as well as the adaptation of that work to the affordances of this specific lithology and boulder (for other examples see [45]).

Secondly, from an iconographic point of view, the presence and centrality of the body of the warrior relate this stela to cases known from other regions where warrior stelae are known. These include the middle Tagus (Aldeanueva de San Bartolomé in Toledo) and Guadiana (Esparragosa de Lares 1, Magacela, and Fuente de Cantos in Badajoz) basins, especially the latter, as well as the lower (El Coronil, Écija 5/El Berraco, Écija 2 in Seville) and middle Guadalquivir basin (Montemayor, Cortijo de la Reina 1 in Córdoba). Interestingly, and perhaps not by chance, all this comparanda include horned headgear, like the Cañaveral de León #2 stela. Another trait of the Cañaveral de León #2 stela is that it does not depict a shield, which is otherwise the most frequent motif among Iberian warrior stelae [38], or a chariot but it does include the image of bow and arrow(s). The latter are not very common in the iconography of warrior stelae. They appear in a small number of cases, mostly without shields, such as São Martinho 2 in the middle Tagus basin, Capilla 3, Alamillo, El Viso 1 (w/shield) and six instances located in the middle Guadiana basin, or Torres Alocaz (w/shield), Cuatro Casas/Carmona (w/shield), Montemolín, and Écija #3 in the lower Guadalquivir basin. It is not by chance that some of the closest iconographic parallels for the stela #2 of Cañaveral de León are found, precisely, in the lower Guadalquivir and the middle Guadiana rivers, as Las Capellanías is located within one of the most important corridors connecting these two regions. The idea of the ‘warrior’ reproduced by these locally manufactured visual displays includes variable but standardized combinations of a wide range of motifs related to warfare (sword, shield, spear, headgear, chariot), hunting (bow and arrow, dog), bodily care and dress (brooch, mirror, comb, razor, tweezers), oral traditions (lyres). More generally, the depicted personages and artefacts associated with them were likely associated with the (´globally´ shared) worldview of those communities, including ideas about the otherworld, foundational myths or esoteric knowledge acquired through the involvement of local elites in long-distance connections with the Atlantic and the Mediterranean during the Bronze Age and the beginning of the Iron Age (c. 1400/1250–750 BC) [11,69,70].

Thirdly, the fact that the personage depicted in stela #2 displays six toes on each foot is highly significant, as it lends support to the hypothesis that stelae were intended to provide graphic accounts of orally transmitted myths, which included special characters, such as heroes [11,34]. Indeed, stela #2 at Las Capellanías might not be the only case where this anatomical feature is found. Other warrior stelae, such as Pedro Abad or Montemolín in the Guadalquivir valley show possible cases of polydactyly in the hands and feet, respectively, although this will require confirmation through high-resolution digital renderings of the motifs. The carvers of a significant group of warrior stelae and stelae with headdress paid great attention to the depiction of fingers and toes. These were often depicted in detail and oversized, suggesting that they were important and meaningful features in the characterisation of the personages depicted. Polydactyly is a rare occurrence in the archaeological record, but there is suggestive evidence that among prehistoric societies it was considered a trait associated with ‘special’ people. To our knowledge, the oldest such case documented in Europe is a female individual found in the richly furnished Copper Age *tholos* of Montelirio, located in the Valencina mega-site [71,72], barely 90 km to the south-east of Cañaveral de León. This trait may well have marked this individual as a special person during her life [72]. Evidence of the significance of polydactyly as a ‘sacred’ physical trait is found in several regions of the world, particularly in Central and North America [73–79].

Finally, there is the matter of the chronology yielded by the two OSL dates obtained from samples of soil lying directly below stela #2. These are the first direct numerical ages ever obtained to date these stelae, with the possible exception of a radiocarbon date from Pocito Chico [27,28] obtained from non-articulated animal bone found as part of the infill of a hut, where the stela had been reused as part of the fabric of a wall. Before the excavation of Las Capellanías, the conventional view about the chronology of ‘warrior’ stelae, placing them largely between the end of the Middle Bronze Age and the beginning of the EIA [28], was drawn from the interpretation of the motifs engraved on them (which, in some cases are recognisable while in others sketchy and unrecognisable), and from the thin (and vague) information that was available as to their context. Stela #2 from Cañaveral de León broadens and enriches this old debate, as the OSL chronology provides maximum ages for its decommissioning, which could have taken place any time between c. 2340 and 1680 BC or later. While running in part counter the conventional wisdom regarding these monuments, an EBA/MBA (first half of the 2nd millennium BC) burial for this stela cannot be ruled out. Firstly, the morphology of the mound into which it was embedded and the cist it was laid next to (Structure 1) are compatible with an EBA chronology. Secondly, its iconography lacks some of the most clearly definable LBA motifs of ‘warrior’ stelae: the two-wheeled chariot and the shields. These are, on the other hand, absent from other warrior stelae dated by some to the LBA or the IA (e.g., Capilla 3, Montemolín) [10]. The only clearly recognisable items in the panoply of stela #2 are a sword and a bow (possibly including a second bow). Swords are a well-known feature of ‘Alentejan’ stelae, which are dated to the EBA/MBA [10,51], and they also appear in EBA burials, particularly in El Argar culture of the Iberian south-east, starting from c. 1800/1700 BC. Therefore, the research undertaken at Las Capellanías opens the possibility that some ‘warrior’ stelae of the Iberian south-west may have been created earlier than previously thought, thus expanding the time range for these remarkable monuments.

However, while a chronology for the decommissioning of stela #2 during the first half of the 2^nd^ millennium BC cannot be ruled out, it is important to note that the infill of soil under the stela was moderately loose and may have been affected by post-depositional alterations. Also, as noted above, because of the nature of the sampling conducted, the samples analysed may have contained grains with different degrees of optical resetting. It is important to reiterate that the OSL results indicate that the process of burial of the soil surface resulting from the deposition of the stela occurred during or following the date range obtained (i.e., 2340–1680 BC; +/- 1σ). Future scientific dating (OSL or otherwise) will be able to refine the temporality of these monuments. This underlines the need for further high-resolution excavation and scientific dating of contexts associated with such stelae, particularly in those cases where clear LBA motifs, such as the two-wheeled chariot, are absent.

## Conclusion

The discovery of stela #2, of the ‘warrior’ type, at the Las Capellanías burial complex, in Cañaveral de León, in June 2022, and its subsequent study, represents a ground-breaking push in the hundred-year-old study of Iberian stelae. For the first time ever, one such stela has been discovered in a primary context in the course of an archaeological excavation, which grants the possibility of controlled, high-resolution and well-contextualised scientific observations regarding its position, chronology and socio-cultural context. In summary, stela #2 was embedded inside the mound of a large burial structure akin to those dating to the Bronze Age across the region, in close association with a large cist and a large block of milky quartz. In addition, it stood on the verge of what today is, as was in recent historical periods, a pathway connecting the lower Guadalquivir valley and the middle Guadiana basin, one of the most important communication routes in pre-modern Iberia. Therefore, the Cañaveral de León #2 stela settles once and for all the decade-old debate concerning the context of these monuments: this example suggests that they operated *both* as burial and landscape markers. In Iberia, the association between burial monuments and pathways in Iberia is well attested since the Late Neolithic and Copper Age [80,81], and the evidence retrieved at Las Capellanías suggests that this was also the case in Bronze Age and Iron Age.

In addition to this, the stelae now discovered at Las Capellanías (three in total, after the September 2023 excavation season) confirm the co-existence and synchronicity of a diversity of stelae often regarded as different types representing different social roles or even traditions of partially diverging chronologies or/and social backgrounds. Stelae which often have been seen as separate in kind and temporality, such as the those ‘with headdress’ (or ‘diademadas’) and those termed ‘warrior’ ones, are found barely a few meters apart at Las Capellanías (Fig 4), near, or integrated in, burial monuments that are adjacent or even attached to each other. This opens an entirely new way of looking at these monuments, presided by the fluidity of graphic concepts, themes and narratives. Those stelae previously referred to as ‘diademated’ and ‘warrior’ can no longer be seen as separate or opposite. This was already suggested by the discovery of the Almadén de la Plata stela #2 in 2005 [35] and is now confirmed by the Cañaveral de León #1, #2 and #3 stelae. The so-called ‘warrior’ and ‘diademated’ stelae were part of a wider system of graphic expression integrating multiple elements in a complex flow of concepts and meanings.

The detailed analysis of the Cañaveral de Leon stela #2 and its context affords new potential ways to look at these monuments. Two significant observations stand out: first, like stela #1 found in 2018, stela #2 was hardly weathered and showed no traces of lichens, which suggests that it was either never placed on an open-air location or was so for a very short time. Secondly, while the smoothing of the slab was thorough and careful in both stelae (Fig 4), the graphic motifs on stela #2 appear to have been engraved rather hastily, thus resulting in a ‘sketchy’ finish. Thirdly, the stela was not found in a ‘Pompeian’ position, but, instead, carefully placed upside down and bottom-up, and embedded inside the stony infill of the mound which cannot have happened by chance. Combined, these three observations suggest the possibility that some stelae were never intended to be erected for long periods of time as enduring monuments but, instead, carved opportunistically and used over a relatively short time span, as part of the funeral practices [70]. That would certainly have been the case of the Cañaveral de León #2 stela, perhaps raised side by side with Structure 1a, the cist standing next to it, and then, shortly after its primary (funerary) use, placed inside the burial mound where it was subsequently found. The same sequence could also be suggested for the Setefilla stela, mentioned above. This counters the largely unchallenged notion, held for more than a century, that all Iberian ‘warrior’ stelae were carefully created, perhaps by specialists, and placed in a standing position, to be seen and kept as enduring monuments. Furthermore, this would fit a broader pattern that is being revealed by the recent re-examination of ‘warrior’ stelae, which shows that some stelae were rather improvised creations that could be engaged with and re-carved shortly after their initial creation [45,69], while others made of very hard lithologies (i.e., silicate quartz-sandstone) from the Zújar region could have been manufactured in a planned manner by specialists during the latest phase of the warrior stelae phenomenon [82].

Finally, the OSL dates obtained suggest that this stela was buried after c. 2700 BC. This opens up the possibility of earlier dates than previously assumed for the creation and reuse of some warrior stelae. While caution must be applied, as these are the first direct OSL dates for such stelae, and, as noted above, a possibility remains that the soil samples included quartz grains associated with an earlier depositional process, this opens up an entirely new line of thinking about the temporality of these remarkable monuments, in which scientific dating reveals itself to be of the essence. Given that the empirical basis on which the chronology of these monuments has been built has traditionally lacked scientific dating, much attention should be paid to this problem in the future.

Altogether, the site of Las Capellanías provides striking new evidence that is set to cause a true upheaval in how Iberian stelae have been approached to this date. Stela #2, together with the new evidence retrieved in the excavations undertaken in June 2022 and September 2023, currently under study, including stela #3, found on top of a burial pit with a substantial amount of cremated human bone and decorated with a striking ‘combination’ of elements from the (hitherto) differentiated ‘diademated’ and ‘warrior stelae, is good proof of how much the long-standing thinking about these monuments is about to be challenged.

## Supporting information

S1 Fig Orthoimage of Structure 1. (JPG)

S2 File Luminescence dating report. (PDF)
