# Supplementary figures and images for: Shedding new light on the context and temporality of Iberian warrior stelae: The Cañaveral de León 2 Stela and Las Capellanías burial complex (Huelva, SW Spain)
Source: PLoS One. 2025 Apr 23;20(4):e0321080. doi: 10.1371/journal.pone.0321080 (PMC12017488; doi:10.1371/journal.pone.0321080)

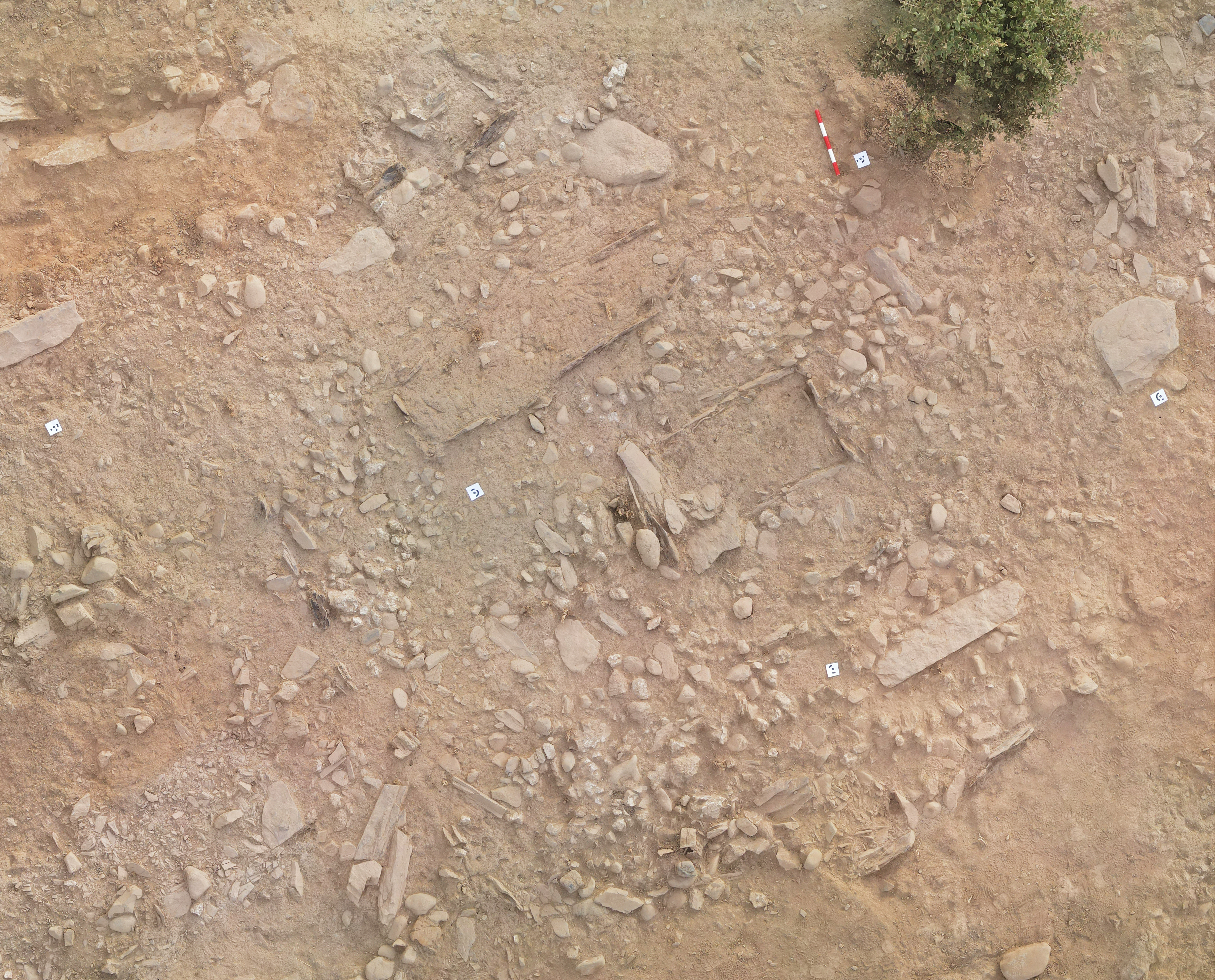

Supplement: S1 Fig — (JPG) [file pone.0321080.s001.jpg]
