# Supplementary material for: Shedding new light on the context and temporality of Iberian warrior stelae: The Cañaveral de León 2 Stela and Las Capellanías burial complex (Huelva, SW Spain)
Source: PLoS One. 2025 Apr 23;20(4):e0321080. doi: 10.1371/journal.pone.0321080 (PMC12017488; doi:10.1371/journal.pone.0321080)
Supplement: S2 File — (PDF) [file pone.0321080.s002.pdf]

LUMINESCENCE DATING REPORT – Warrior stela

Methodology

Sampling and sample preparation

The OSL samples LC22UEC1OSL1 and LC22UEC1OSL2 were given lab IDs, DLL24s179 and DLL24s180 respectively. They were prepared under subdued red-light conditions in the Durham University Luminescence Laboratory (DLL). The outer, potentially light-exposed, portions of each sample were removed and used for environmental dose rate measurements and estimation of the sample moisture content. Additional samples were obtained from: from the stones lying underneath the OSL samples, and from granite with petrographic characteristics similar to those identified in the granite of the stela located above the samples to provide data for calculation of the dose rates.
The sampled sediments were treated with hydrochloric acid (1M HCl) and hydrogen peroxide (H_2O_2) to remove carbonate and organic matter respectively. The samples were dry sieved to isolate the 150-200 µm sediment fraction sizes. Quartz was then extracted from the selected sediment fraction size by using density separations at 2.62 and 2.70 g/cm^3 and a subsequent HF acid etch (23M HF for 40 minutes, followed by a 10M HCl rinse). The samples were then re-sieved at 150 µm to remove acid-soluble fluorides and any grains that had been significantly reduced in size by etching.

Luminescence measurements

Equipment

Luminescence measurements were performed with a TL/OSL-DA-20 Riso reader equipped with a calibrated ^{90}Sr/^{90}Y source for administering radiation dose. Optical stimulation of multi-grain aliquots was carried out using either a blue (470 nm) or an infrared (875 nm) light emitting diode (LED) array. The luminescence emissions were detected by an ET Enterprises PDM9107Q photomultiplier after passing through Hoya U-340 filters (7.5 mm thickness).

Equivalent dose determination

Equivalent dose (D_e) determinations were carried out using a single-aliquot regenerative-dose (SAR; Murray and Wintle, 2000, 2003) procedure with 9 mm multi-grain aliquots containing on average ca 2k grains. 22 replicates were analysed for samples DLL24s179 and DLL24s180.

A five-point SAR protocol was used to bracket the expected paleodose with an additional recycling point to check for uncorrected sensitivity changes. A number of additional regeneration points were also included to monitor the quality of the data generated: (1) a zero dose point to measure recuperation and thermal transfer; (2) a repeat measurement of the initial regeneration dose to calculate the recycling ratio, which tests the internal consistency of the dose response curve and thus the applicability of the SAR protocol; (3) a second repeat of the initial regeneration dose followed by a room temperature IR bleach and subsequent OSL measurement to calculate the IR depletion ratio, which allows contaminating feldspar grains to be detected (Duller, 2003). Preheat temperatures were determined using dose recovery preheat plateau tests (Murray and Wintle, 2003). Following these preheat regimes tests, a preheat treatment of 220°C for 10s followed by a second preheat of 160°C for 10s was adopted.

Dose recovery tests were performed by applying a given dose of 12 Gy to aliquots of sample DLL24s179. The recovered doses were within 10% of the given dose, demonstrating that the protocol used successfully measured the known irradiation dose administered prior to any thermal treatment.

The dose response curve obtained for each measured disc was fitted with a saturating exponential plus linear function. The D_e values were calculated by projecting the sensitivity-corrected natural luminescence intensity onto the dose response curve, where the luminescence signal comprised the initial 0.12 s of the OSL decay curve, and the last 0.4 s of the signal was subtracted as the background signal. The standard error associated with each individual D_e determination was estimated by Monte Carlo simulation. Curve fitting, D_e determination and Monte Carlo simulations were performed using version 4.31.9 of the Luminescence Analyst software (Duller, 2007).

The D_e values calculated were only accepted where: (1) the natural signal could be distinguished from the background signal (determined using Luminescence Analyst 'sig. >3 sigma above BG' rejection criterion); (2) the recycling ratio was within 10% of unity; (3) the recuperation measured during the zero dose test was lower than 5%; (4) the IR-depletion ratio was lower than two standard errors below unity; (5) the standard error on D_e was less than 40%.

Dose rate determination

The beta and gamma dose rate to each OSL sample due to the sediment and the granite stella slab were estimated based on the ICP-MS & AES measurements of parent uranium and thorium, potassium and rubidium concentrations in samples of these media. The latter were converted to infinite medium dose rates using factors calculated by Guérin et al. (2011). The gamma dose rate within the OSL sampled volumes due to lithics lying under the OSL samples was calculated (see below) using the concentrations of the lithogenic radionuclides measured with a high-resolution germanium gamma spectrometer. The beta dose rate was corrected for: i) 150-200 µm grain size attenuation factors from Guérin et al. (2012), and an etch attenuation factor after Bell (1979). The dose rates were corrected for an estimated water content of 5% with an associated error of 2% to account for seasonal variations and past changes in the sediment. Measured water contents were used for the lithic samples (granite and stones). Cosmic dose rates were calculated using the site altitude / coordinates and the burial depth of the samples assuming an overburden density of 1.8 g/cm^3 for sediment, 2.4 g/cm^3 for stone and 2.8 g/cm^3 for the granite slab.

Dose rate modelling

The gamma dose rate was calculated taking into account the variation in radioactivity within the burial medium to a distance of ca 30 cm from each OSL sample location. This was determined by calculating the gamma dose at the OSL sample location due to each of the contributing layers (Fig. 1), incorporating their density, water content, layer thickness, and distance from the OSL sample, following the superposition model given in Aitken (1985) that is based on the calculations of Løvborg. The simplified model shown in Fig. 1 represents a 2D version of the sampled section, with the granite stele slab as the top layer, the sediment layer from which the sample was taken as the target layer, and with the lower layer consisting of stones. Although only one example of the latter was received for testing, their contribution to the gamma dose rate is relatively small (Fig. 1). The sample offset within the target layer was also considered (Figure 1).

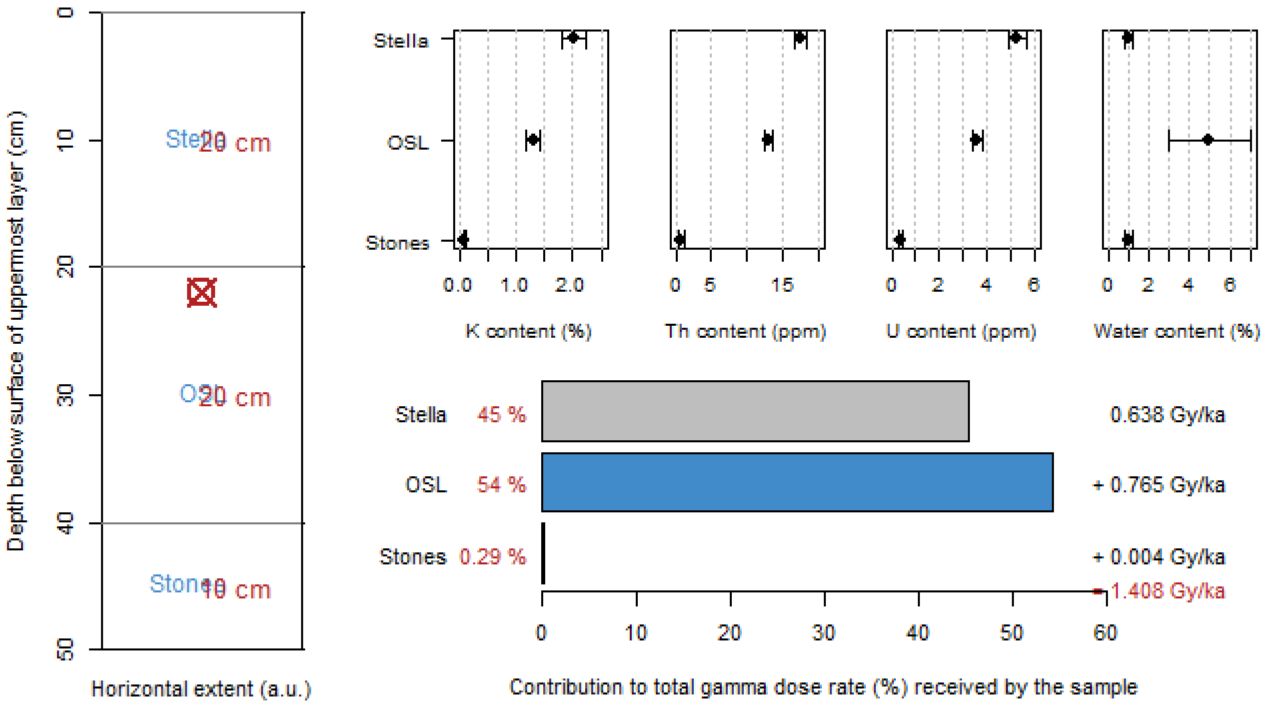

Figure 1: Results of the 2D model used to calculate the contribution to the total gamma dose rate received by the sample. The depth of both samples is indicated in the modelled section by the crossed circle.

Results / Discussion

Samples DLL24s179 and DLL24s180 exhibited low overdispersion (OD, respectively 18 and 17%(calculated using the R Luminescence package) which indicates that they were optically reset at deposition and also sampled in a homogeneous layer affected by little to no bioturbation. The D_e distributions are illustrated in the abanico plots (Figs 2 and 3).

The OD, skewness, and kurtosis of each D_e distribution were analysed to identify the most suitable age model. The skewness indicates a slight bias towards smaller values, but the data still fall within acceptable limits to be considered a 'normal' distribution and the Central Age Model (CAM) was adopted to calculate the burial dose (D_b) for each sample. The values of dose rate, burial dose (D_b), and age are summarised in Table 1 for each sample.

There are also a few considerations regarding the reliability of these ages:

1. **Sample Collection & Mixing** – The abanico plot for Sample 1 shows a slight skew towards younger De values, which, though subtle, could suggest some mixing. One possibility is that this skew reflects the incorporation of a thin layer of sediment disturbed during the excavation of the mound to bury the stela. Grains reset during this process may have been partially mixed into the sampled material. Since only the uppermost millimetres of sediment would be fully bleached and capable of dating this event, but sampling covered a 2 cm depth. Alternatively, the presence of younger grains could result from intrusive processes such as bioturbation, root activity, or beta microdosimetry effects. However, these grains are present in such small quantities that they do not appear to have significantly influenced the final age calculation.
2. **Dose Rate Assumptions** – Ideally, for complex burial environments like this one, we would measure gamma dose rates in situ, given their influence over a ~30 cm radius. However, this was not possible, so we had to rely on a simplified 2D three-layer dose rate model following Aitken (1985). While reasonable within the timeframe of this project, this model involves several key assumptions:

- **Gamma Radiation Contribution** – The gamma dose rate from the stones found on site was estimated from a single stone sample, while multiple stones of different compositions surround the sample. We had to assume that the analysed stone is representative of all others, which is a significant simplification.
- **Stela Composition** – The stela's contribution to the dose rate was estimated using a granite sample from a "similar" source, but this is not from the stela itself. While it may be the same material, this remains an assumption.
- **Variability of Dose Rates across different lithologies** – The actual stratigraphy at the site is complex, but our model is highly simplified and does not fully capture this variability. The stela is deposited at an angle, whereas our model assumes a planar geometry. This discrepancy affects both cosmic dose rates and gamma dose distribution, but given the available time and constraints, a more refined model was not feasible.

Given these factors, while the OSL date is a robust estimate for the age of the mound, it should be interpreted cautiously in relation to the stela. It provides a maximum age for its deposition, but without an upper constraint, other kinds of evidence are needed to ascertain when the stela was actually placed there.

Sample	*Lab ID*	*Environmental Dose Rate (Gy/ka)*	*D_b (Gy)*	*Age (ka)*
LC22 UE1C OSL0001	*DLL24 S.179*	*3.32 ± 0.24*	*13.08 ± 0.46*	*3.94 ± 0.32*
LC22 UE1C OSL0002	*DLL24 S.180*	*3.32 ± 0.24*	*13.36 ± 0.50*	*4.03 ± 0.33*

*Table 1**: Burial doses (D_b), Dose rates and Ages for the analysed samples.** The measurements were performed with quartz 9 mm multi-grain aliquots each containing ca. 2k grains. The dose rates were modelled and corrected for measured water content, grain size and etch attenuation factors (see dose rate determination sub-section for details). The quartz grains were assumed to contain negligible concentrations of lithogenic radionuclides. The ages are given with an uncertainty of ±1σ.*

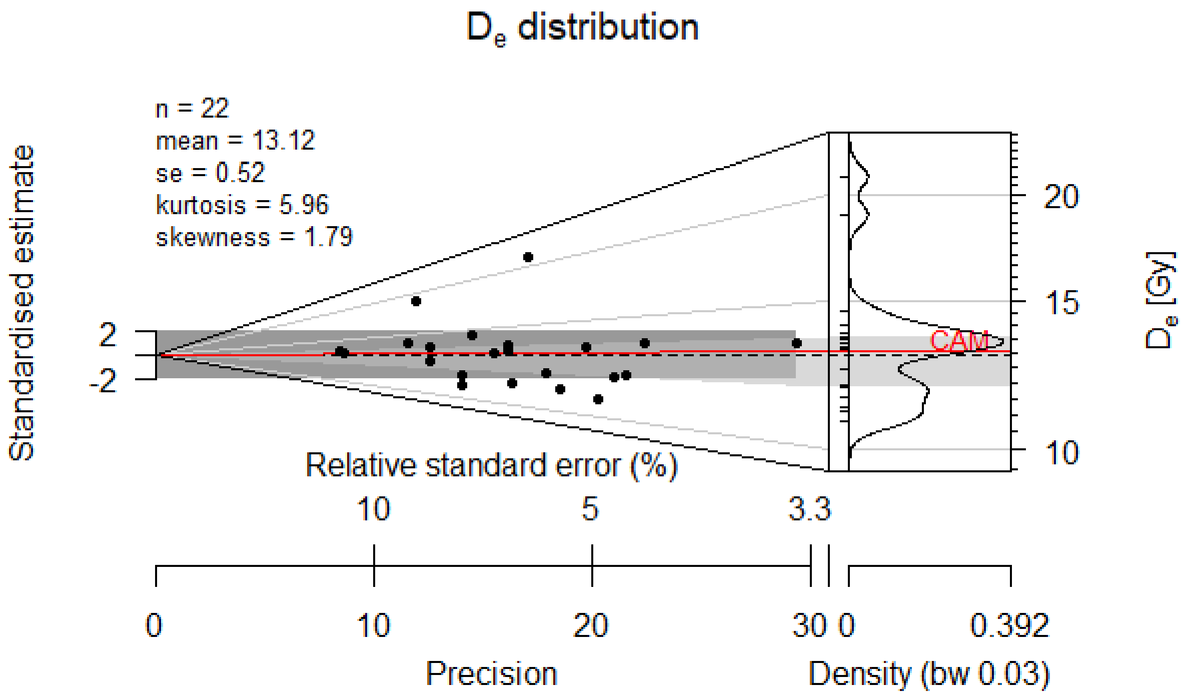

Figure 2: Abanico plot of D_e values for sample DLL24s179. The dotted line shows the weighted mean, $\overline{D}_e$, with the dark grey shaded area showing the values within a 2σ range. The light-grey shaded area shows the scatter polygon highlighting D_e values within the quartile range.

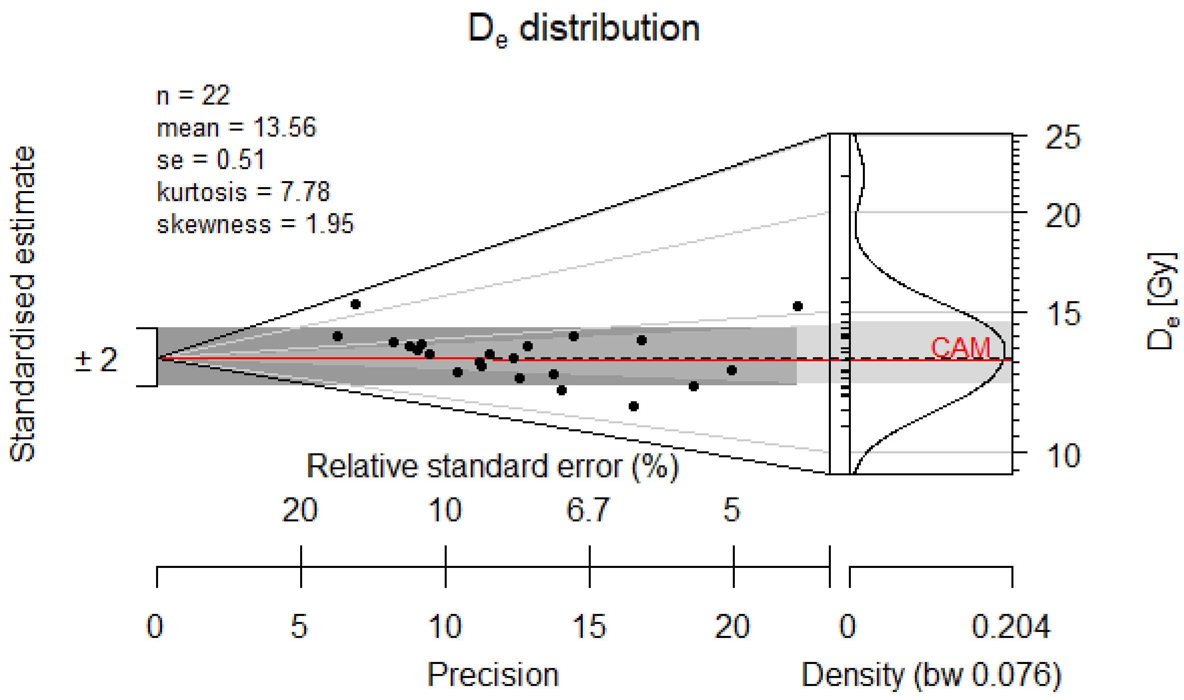

Figure 3: Abanico plot D_e values for sample DLL24s180. The dotted line shows the weighted mean, $\overline{D}_e$, with the dark grey shaded area showing the values within a 2σ range. The light-grey shaded area shows the scatter polygon highlighting D_e values within the quartile range.

REFERENCES

Aitken, M.J. (1985). Thermoluminescence dating. Academic Press, London.
Bell, W.T., 1979. Attenuation factors for the absorbed radiation dose in quartz inclusions for thermoluminescence dating. Anc. TL 8, 1-12.
Duller, G.A.T., 2003. Distinguishing quartz and feldspar in single grain luminescence measurements. Radiat. Meas. 37, 161–165.
Duller, G.A.T., 2007. Assessing the error on equivalent dose estimates derived from single aliquot regenerative dose measurements. Anc. TL 25, 15-24.
Galbraith, R., Laslett G., 1993. Statistical models for mixed fission track ages. Nuclear Tracks and Radiation Measurements 21: 459–470.
Guerin, G., Mercier, N., Adamiec, G., 2011. Dose-rate conversion factors: update. Anc.TL 29, 5-8.
Guerin, G., Mercier, N., Nathan, R., Adamiec, C., Lefrais, Y., 2012. On the use of the infinite matrix assumption and associated concepts: a critical review. Radiat. Meas. 47, 778-785.
Murray, A.S., Wintle, A.G., 2000. Luminescence dating of quartz using an improved single-aliquot regenerative-dose protocol. Radiat. Meas. 32, 57–73.
Murray, A.S., Wintle, A.G., 2003. The single aliquot regenerative dose protocol: potential for improvements in reliability. Radiat. Meas. 37, 377–381.
